# Impact of Facial Conformation on Canine Health: Brachycephalic Obstructive Airway Syndrome

**DOI:** 10.1371/journal.pone.0137496

**Published:** 2015-10-28

**Authors:** Rowena M. A. Packer, Anke Hendricks, Michael S. Tivers, Charlotte C. Burn

**Affiliations:** 1 Department of Clinical Science and Services, Royal Veterinary College, Hatfield, Hertfordshire, United Kingdom; 2 School of Veterinary Sciences, University of Bristol, Langford House, Langford, Bristol, BS40 5DU, United Kingdom; 3 Department of Production and Population Health, Royal Veterinary College, Hatfield, Hertfordshire, United Kingdom; Faculty of Animal Sciences and Food Engineering, University of São Paulo, BRAZIL

## Abstract

The domestic dog may be the most morphologically diverse terrestrial mammalian species known to man; pedigree dogs are artificially selected for extreme aesthetics dictated by formal Breed Standards, and breed-related disorders linked to conformation are ubiquitous and diverse. Brachycephaly–foreshortening of the facial skeleton–is a discrete mutation that has been selected for in many popular dog breeds e.g. the Bulldog, Pug, and French Bulldog. A chronic, debilitating respiratory syndrome, whereby soft tissue blocks the airways, predominantly affects dogs with this conformation, and thus is labelled Brachycephalic Obstructive Airway Syndrome (BOAS). Despite the name of the syndrome, scientific evidence quantitatively linking brachycephaly with BOAS is lacking, but it could aid efforts to select for healthier conformations. Here we show, in (1) an exploratory study of 700 dogs of diverse breeds and conformations, and (2) a confirmatory study of 154 brachycephalic dogs, that BOAS risk increases sharply in a non-linear manner as relative muzzle length shortens. BOAS only occurred in dogs whose muzzles comprised less than half their cranial lengths. Thicker neck girths also increased BOAS risk in both populations: a risk factor for human sleep apnoea and not previously realised in dogs; and obesity was found to further increase BOAS risk. This study provides evidence that breeding for brachycephaly leads to an increased risk of BOAS in dogs, with risk increasing as the morphology becomes more exaggerated. As such, dog breeders and buyers should be aware of this risk when selecting dogs, and breeding organisations should actively discourage exaggeration of this high-risk conformation in breed standards and the show ring.

## Introduction

The domestic dog is the most morphologically diverse terrestrial mammalian species known to man [1]; however, pedigree dogs are strongly selected for aesthetics dictated by formal Breed Standards, and breed-related disorders linked to conformation are ubiquitous and diverse [2,3]. Artificial selection for desired traits in domestic animals can cause unintended changes in other traits, which can be pathological, e.g. lameness in highly productive dairy cows [4] and broiler chickens [5]. The mechanisms underlying such pathologies include the inadvertent genetic consequences of inbreeding or linkage disequilibrium [3], or may be direct physical consequences of the desired trait [2]. Brachycephaly, or foreshortening of the facial skeleton, is a discrete skeletal mutation [6] where altered growth of the basioccipital and basisphenoid bones manifests in a shortening of the basicranial axis [7]. This results in the characteristic short-muzzled, or flat facial conformation that has been intensely selected for by dog breeders to develop many popular companion dog breeds, due to the anthropocentric appeal of their juvenile, human like features [8]. Despite the increasing popularity of brachycephalic breeds such as the Pug, Bulldog and French Bulldog in the UK and internationally [9], this conformation is not benign and is associated with several inherited disorders of the head and neck [2,10].

### Anatomical abnormalities

Brachycephalic Obstructive Airway Syndrome (BOAS) is a debilitating respiratory syndrome that predominantly affects brachycephalic dogs, whereby soft tissue blocks the airways during respiration (Video A in S3 File). A previous study of this disorder dichotomised breeds as brachycephalic or not, and showed that 39 of their 45 BOAS cases occurred in brachycephalic breeds, with brachycephaly conferring an odds ratio of 38 on the risk of BOAS [11]. The condition arises in brachycephalic animals because, despite a marked reduction in facial skeleton length [7], the soft tissue structures of the oral cavity (e.g. soft palate, tongue, tonsils) are not proportionally reduced [12]. As an affected dog matures, the compacted soft tissue increasingly impede airflow by blocking the larynx and nasopharynx, and impairs the thermoregulatory function of the nose via internal [13] and external nasal obstruction [14]. Within the nose, turbinate growth in young brachycephalic dogs continues despite inhibition of growth of the mid-face, resulting in relatively oversized turbinates [15]. The resulting contact between turbinate lamella mucosal surfaces impedes nasal airflow. Externally, the wing of the nostril (ala nasi) is congenitally deformed in many brachycephalic dogs [14], with a narrowing of the nostrils (‘stenotic nares’). These primary abnormalities can result in markedly increased respiratory efforts to overcome airway resistance, fostering collapse of the airway [16], most commonly the larynx [17,18]. Laryngeal collapse is the most common and serious secondary change associated with BOAS, with a guarded prognosis for late stage laryngeal collapse [19,20]. The functional problems subsumed under the term BOAS are thus the result of the skeletal shortening and the relationship between the facial skeleton and the soft and hard tissue structures contained within it. Dogs of breeds not traditionally classified as brachycephalic have rarely been diagnosed with BOAS in previous studies, such as the Chow Chow, Rottweiler and Pomeranian [21]. These breeds may be classed as mesocephalic (a head of medium proportions); however, without morphometric data it is not possible to ascertain whether these individuals were short-muzzled for their breed, or whether other risk-factors for BOAS were present.

### Clinical signs and welfare impact

BOAS is characterised by a chronic shortness of breath and subsequent difficulties in exercising (e.g. walking, running and playing), a propensity to overheating, increased and abnormal respiratory noise (e.g. snoring and snorting), low blood oxygen levels and consequently, collapse [22]. BOAS affected dogs are prone to heat stroke, which can result in death [23]. Severely affected individuals exhibit laboured breathing, often adopting a wide stance with their elbows abducted from their chest, with the use of accessory abdominal musculature [24] and over inflation of the chest [25] observed. BOAS has been a recognised disorder for many years, with surgical techniques developed to treat this syndrome described as early as the 1940’s [26,27].

BOAS has potentially severe welfare consequences [28], with the most affected dogs described as having “*little or no activity”* because they are fully occupied just with breathing [29]. Any form of stress, exercise or excitement can cause severe respiratory distress in such dogs, and occasionally even death [16,17] Minor aggravations can lead to severe respiratory distress [23], with arousal by both negative and positive experiences (e.g. stress, but also exercise and excitement) acting as aggravators [23,25]. Clinical signs of BOAS can be evident whilst the dog is awake or asleep, with audible snoring reported in 100% of BOAS affected dogs vs. 21% of unaffected dogs in a recent study [22], and sleep-disordered breathing (including episodes of ‘apnoea’, cessation of breathing) well researched in the Bulldog [30]. The effects of BOAS are not just limited to the respiratory system, with chronic negative pressure in the chest cavity leading to gastrointestinal tract lesions, manifested as clinical signs such as gagging, regurgitation and vomiting [31]. Clinical signs are often severe by 12 months of age [32] and are life-long thereafter.

### Conformation and disease

Despite the name of the syndrome, scientific literature quantitatively linking brachycephaly with BOAS is lacking, with this hypothesised relationship based on overrepresentation of brachycephalic breeds in international case series of BOAS (e.g. [11]). BOAS has been reported in over 10 brachycephalic breeds internationally [22]; however, brachycephaly is not a binary trait. Instead, relative muzzle length is a highly variable aspect of canine skull morphology [33]. International inquiries regarding dog breeding practices [34,35] have called for information quantifying the extent to which BOAS relates to craniofacial conformation, and therefore what constitutes a muzzle being ‘too short’, which was previously unknown. These quantitative limits were initially proposed by the Council of Europe [35], where it was suggested that “*Maximum values for the shortness of skull*, *respectively the nose to avoid breathing difficulties*” should be set.

### Breed standards

In recent surveys of veterinarians [36] and other canine stakeholders in the UK [37], altering breed standards was the most common suggestion to reduce the prevalence of inherited diseases in pedigree dogs, with veterinarians also strongly disagreeing with the statement that breed standards support the health and welfare of dogs [36]. Data linking inherited diseases with morphologies encouraged by breed standards could thus encourage the revision of breed standards internationally, with quantative limits included to encourage the necessary changes and reverse the current trend towards ‘short’ muzzles, as evidenced in current breed standards (Table 1), if this morphology does indeed increase BOAS risk.

**Table 1:** Kennel Club and American Kennel Club breed standards of popular brachycephalic breeds, describing ‘short’ muzzles which may put them at risk of BOAS. The ‘nose’ refers to the nose leather, but can constitute a large proportion of the muzzle length in brachycephalic dogs.

	Breed standard text referring to the relative length of the ‘muzzle’, ‘face’ and/or ‘nose’	
Breed	Kennel Club	American Kennel Club
Pug	Muzzle relatively **short**, blunt, square [61]	The muzzle is **short**, blunt, square, but not upfaced [64]
French Bulldog	Nose relatively **short** [63]	The muzzle broad, deep and well laid back. The stop well defined, causing a hollow groove between the eyes with heavy wrinkles forming a soft roll over the extremely **short** nose [65]
Bulldog	Face relatively **short** [62]	The face, measured from the front of the cheekbone to the tip of the nose, should be extremely short, the muzzle being very **short**, broad, turned upward [66]
Pekingese	Muzzle must be evident, but may be relatively **short** and wide [75]	Muzzle—It is very flat, broad, and well filled-in below the eyes. Nose—It is broad, **short** and black.[76]
Griffon Bruxellois	Relatively **short**, wide muzzle [77]	Nose very black, extremely **short**, its tip being set back deeply between the eyes so as to form a lay-back.[78]
Japanese Chin	Muzzle **short**, wide [79]	Muzzle—**short** and broad with well-cushioned cheeks and rounded upper lips that cover the teeth. Nose—very **short**. [80]
Boston Terrier	Muzzle relatively **short**, square, wide [81]	The muzzle is **short**, square, wide and deep and in proportion to the skull. It is free from wrinkles, shorter in length than in width or depth; not exceeding in length approximately one-third of the length of the skull. [82]
Shih Tzu	Muzzle of ample width, **square**, short [83]	Square, **short**, unwrinkled, with good cushioning, set no lower than bottom eye rim; never downturned. Ideally, no longer than 1 inch from tip of nose to stop, although length may vary slightly in relation to overall size of dog. [84]

### Aims

The aim of this study was to confirm and quantify the extent to which the brachycephalic craniofacial conformation is associated with BOAS risk, and whether more extreme brachycephalic conformations are at a higher risk of BOAS than moderate or mildly brachycephalic morphologies. This is with the practical aim of being able to provide data upon which to base quantitative recommendations regarding the degree of brachycephaly that is ‘acceptable’ to breed for, helping reduce risk of this disorder. This study deliberately used a morphometric methodology that is non-invasive and easily applicable to conscious dogs without the need for special equipment. In addition, we aimed to investigate whether BOAS is related to craniofacial conformation *per se* or some other aspect of breed affiliation, e.g. genetic background or lifestyle factors. We did this by first examining the relationship between conformation and BOAS in all breeds and cross breeds, and then limiting the analysis solely to affected breeds, to see if any relationship remained within affected breeds.

### Study Plan

To investigate how craniofacial morphology related to respiratory function, we examined the conformation and clinical status of two populations of dogs; firstly, dogs of any breed or crossbreed entering a veterinary referral hospital for any condition (referred to as ‘Study 1’) to gain general estimates of the relationship between BOAS and muzzle length in a large, genetically and morphometrically varied population of domestic dogs. This was followed by studying a second population outside the referral hospital environment (referred to as ‘Study 2’), this time focussing on brachycephalic dogs only, to test the robustness of the initial estimates generated in Study 1.

## Materials and Methods- Study 1

### Morphometrics

**Fig 1:**
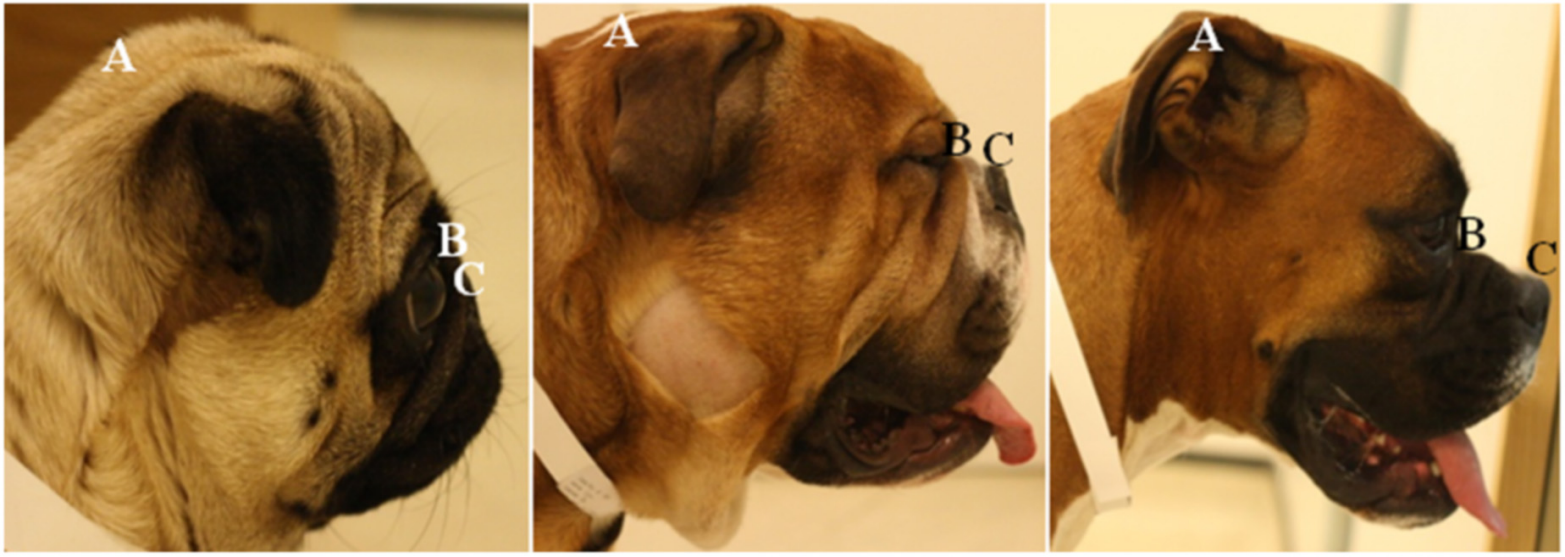
Diagram of how to measure (i) cranial length (A-B) and (ii) muzzle length (B-C). Measurements were taken using a soft measuring tape. Cranial length is defined as the distance (mm) from the occipital protuberance (A) to the stop (B). Muzzle length is defined as the distance (mm) from the dorsal tip of the nasal planum (C) to the stop (B). The precise locations of the nasal planum, stop, and occipital protuberance are determined through palpation as well as visually, but the lettering indicates their approximate locations on the photographs. This is demonstrated in (left-right) an extremely brachycephalic Pug (CFR = 0.08), a moderately brachycephalic Bulldog cross (CFR = 0.23) and a mildly brachycephalic Boxer (CFR = 0.35).

The conformation of all dogs was measured using established breed-defining measurement protocols [38]. Thirteen conformational features shown to be breed-defining were measured: muzzle length, cranial length, head width, eye width, neck length, neck girth, chest girth, chest width, body length, height at the withers and height at the base of tail, fore limb circumference and hind limb circumference (all in cm). All measurements were made to the nearest millimetre. Muzzle length was defined as the distance (mm) from the dorsal tip of the nasal planum to the stop, and was measured from the tip of the nose to just between the eyes where the inside corners of the eyes meet (as the stop is not discernible on longer-muzzled dogs with a less pronounced facial angle). Cranial length (CL) was defined as the distance (mm) from the stop to the occipital protuberance, following the curve of the cranial surface (rather than a linear measure), and was measured from just between the eyes up the face, between the ears, to the back of the head where the bony process projects out. Both measurements were taken using a standard 1 m soft measuring tape. The degree of brachycephaly (facial foreshortening) was quantified by the craniofacial ratio (CFR): the muzzle length divided by the cranial length [22] (Fig 1). All dogs were examined for the presence of a nasal fold; defined as a discernible fold of skin on the dorsal surface of the muzzle that was present without manipulation of the skin, and could be easily grasped between vernier callipers; this was recorded as a binary trait.

The craniofacial morphometric parameters used in this study were not those previously created for the examination of dry skulls using callipers to measure linear distances between set points [39,40], or those used in CT/MRI analysis of the skull [41,42]. Instead, the measurements used here were first described in a previous large-scale study (>1000 dogs) of a wide variety of dog breeds in the United States [38], and created with the purpose of being easily replicated by owners in the home; thus these aligned well with our aim of using measurements that breeders and owners can take with simple equipment easily available to them. The craniofacial ratio previously devised by the authors of the current study [22] produces intuitive figures that reflect the degree of foreshortening of the muzzle. Further studies may focus on the inter-rater and intra-rater reliability of this measure.

Weight (kg) was measured in all dogs on regularly calibrated digital scales. Body condition score (BCS) was assessed on a a 9 point scale [43] by a single-rater (RMAP). To test for potential effects of other aspects of body shape and size, as seen in a previous study of conformation-related disease [21], Principal Component Analysis of the remaining measurements was carried out, to attempt to capture two overarching aspects of each dog’s morphology: overall skeletal body size (Principal Component 1: PC1) and the ‘thickness/broadness’ of the dog’s body (Principal Component 2: PC2); components that were previously identified from a similar canine morphometric data set [38]. Low PC1 indicates a small skeletal size (e.g. a Chihuahua) while high PC1 indicates a large skeletal size (e.g. a Great Dane). Low PC2 indicates a narrow and slim-boned body shape (e.g. a Greyhound), while a high PC2 indicates a broad and thick-boned body shape (e.g. a Mastiff). Cranial length and muzzle length were omitted from that analysis, so that these variables were not included in the statistical models twice. Principal components were extracted based on eigenvalue, with only those greater than one extracted.

### Recruitment of dogs

Between December 2010 and January 2012, every dog referred to the Royal Veterinary College Small Animal Referral Hospital (RVC SARH) was considered for inclusion in Study 1. Owners of dogs referred to any clinical service for a routine appointment were approached. All dogs were considered for recruitment prior to their arrival at the hospital, and were excluded on a case-by-case basis if they were:

- Presented for a disorder that would make them unsuited to leaving wards/nursing care during their stay in the hospital, or too painful/uncomfortable to be handled.
- Known to be aggressive and therefore not suitable for handling
- Isolated from the general hospital population for infection control
- Already recruited to a separate clinical trial/study within the hospital (N.B. no other studies investigated respiratory disease, obesity, GI disease or other breed-specific studies were ongoing at this time that may have biased enrolment)

The owners of all remaining dogs (n = 700) were approached in the waiting room before their consultation, to request consent.

### Identification of BOAS cases

Dogs referred to the RVC SARH for BOAS were diagnosed based on clinical history, physical examination, and examination of their upper airway under general anaesthesia. BOAS frequently goes unnoticed by owners because they perceive that its signs are ‘normal’ in their dog or in brachycephalic breeds, being of early onset, highly prevalent and long-lasting [22]. Therefore, to ensure that no dogs were erroneously classed as unaffected, dogs referred to the hospital for unrelated reasons were also assessed for BOAS. Examination of the upper airways under anaesthesia was not feasible for these dogs for ethical and practical reasons, including the financial cost to the owner. Instead, therefore:

1. All study dogs were examined for stenotic nares (narrowed nostrils). This external abnormality is comparatively simple to diagnose compared with the invasive diagnosis of internal airway abnormalities. However, the severity of stenosis normally involves a subjective visual assessment [44]. In some dogs stenosis may be mild, while in others can result in the necessity to almost continually breathe with the mouth open [45]. The ‘nares ratio’ was quantitatively calculated for all dogs, as previously published [22].
2. Questionnaires were given to all owners, with regard to their dog’s behaviour, health and lifestyle. The frequency of respiratory difficulties and the severity of abnormal respiratory sounds were requested in four activity scenarios: (i) at rest, e.g. while lying down awake; (ii) while gently walking, e.g. walking around the house; (iii) during activity/exercising e.g. on a walk, whilst playing; and (iv) while asleep. The degree of owner-reported respiratory difficulty and respiratory noise in the four scenarios was later quantified into a composite score, the “Owner Reported Breathing” (ORB) score, out of 40 [22].
3. Clinical History: To avoid misclassification of cases, all study dogs’ clinical histories including their physical examination findings at the RVC SARH were examined to identify the presence of other respiratory or cardiac disorders that may have contributed to their ORB score.

ORB score and nares ratio values from formally diagnosed dogs were used as a diagnostic cut-off point to classify affected versus unaffected dogs. These thresholds were thus based on the lowest ORB score observed in a formally affected dog (8/40) and the upper value of 95% confidence interval of the nares ratio for the formally affected dogs (0.30; i.e. the widest nares of any formally affected dog). As such, dogs with ORB scores >8 and nares ratio values <0.30 were classed as affected.

### Ethics statement

This study was approved by the Royal Veterinary College’s Ethics and Welfare Committee (URN 2010 1054).

### Statistical analysis

Data were analysed using generalised linear mixed models for binary outcomes in R, using lmer from the lme4 package. Being affected by BOAS was the binary response variable in all models. Relevant morphometric predictors (all individual measures including absolute and relative parameters e.g. absolute muzzle length and CFR), BCS, weight and age were modelled as continuous fixed effects. Neck girth was included as an additional morphometric predictor because it predicts a comparable human respiratory disorder, obstructive sleep apnoea syndrome (OSAS) [46–50]. The presence of a nasal fold (wrinkle) was also investigated as a predictor, in case it was an external manifestation of excess soft tissue internally. The principal component indicative of body size (PC1) was also taken into account in case of a scaling effect on BOAS risk, as has been found with at least one other conformational disorder [21], along with the principal component indicative of skeletal thickness/broadness (PC2). Breed was included as a random effect, with all cross breeds coded plainly as ‘cross breed’ due to the unknown parentage of many of these dogs. This random effect took into account the genetic non-independence of multiple members of the same breed in the study population, and possible demographic and environmental factors. Non-morphometric predictors i.e. signalment: age, sex, neuter status, Parker genetic groups [51] and Kennel Club groupings [9] were tested in all models.

Multicollinearity was checked for in all models, identified from inflated standard errors in the models, and thus avoided. Model fit was assessed using the deviance and Akaike's information criterion. From the model output, equations were used to calculate the probability of being affected by BOAS at different values of CFR, using breed-specific random effects to compare different breeds’ risks. Estimates were only calculated for the CFR range exhibited in that breed, for reasons of biological plausibility. For the variables held constant in the model whilst the fixed effect under investigation was varied, the mean value was used for that breed, to represent an average member of the breed, and BCS was held constant at 5, to represent an ideal weight dog.

## Results–Study 1

### Population demographics and clinical status

In the referral population, 700 dogs represented 97 breeds (this population has been described in detail in Packer *et al*. [21]). All owners consented for their dog to be involved in the study. Most dogs were pure bred (87%), with 40% male neutered, 17% male entire, 32% female neutered and 10% female entire. The mean ± SE age was 5.17 ± 0.13 years, and mean ± SE weight (kg) was 21.5 ± 0.55, with 46% of dogs being overweight (BCS>5/9).

Of the 700 dogs recruited, 70 were categorised as affected by BOAS. Thirty five were formally diagnosed, and a further 35 dogs met both of the non-invasive inclusion criteria. There were no significant differences between formally diagnosed vs. criteria-diagnosed dogs for craniofacial ratio (mean: 0.19, SE: 0.02 vs. mean: 0.15, SE: 0.01, Mann-Whitney U = 508, p<0.05), nares ratio (mean: 0.25, SE: 0.03 vs. mean: 0.18, SE: 0.01, Mann-Whitney U = 496, p>0.05) or breed distribution (*X* ^2^ = 20.8, p>0.05) and thus their results are combined as one ‘affected’ category due to their inherent similarities. Twelve breeds were affected (Table 2). Affected cross breeds included two Bulldog crosses ‘Victorian Bulldogs’, and one Pug x Chihuahua ‘Chug’ and one ‘Pugalier’ (Pug x Cavalier King Charles Spaniel).

**Table 2:** Synopsis of breeds affected by brachycephalic obstructive airway syndrome (BOAS), morphometric risk factors and modelled BOAS probabilities.

Breed	Study 1		Study 2		Study 1		Study 2		Study 1	Study 2
	n	Affected (%)	n	Affected (%)	Median craniofacial ratio (IQR)	Median neck girth (IQR)	Median craniofacial ratio (IQR)	Median neck girth (IQR)	Min-Max predicted BOAS risk	Min-Max predicted BOAS risk
Pug	32	88%	32	91%	0.08 (0.06)	32.2 (4.82)	0.12 (0.06)	31.8 (3.10)	0.69–0.97	0.48–0.95
French Bulldog	13	70%	4	75%	0.19 (0.06)	33.0 (6.25)	0.18 (0.05)	35.3 (3.70)	0.73–0.89	0.30–0.85
Bulldog	16	63%	6	33%	0.22 (0.11)	42.2 (7.58)	0.25 (0.08)	43.8 (9.75)	0.38–0.74	0.26–0.88
Boston Terrier	6	83%	2	50%	0.14 (0.04)	30.2 (2.55)	0.23	28.2	0.21–0.72	-
Japanese Chin	0	-	10	80%	-	-	0.04 (0.06)	23.8 (3.38)	-	0.84–0.96
Pekingese	3	67%	3	0%	0.12	31.3	0.11	28.0	0.50–0.65	0.66–0.79
Dogue de Bordeaux	6	67%	1	0%	0.36 (0.03)	55.5 (9.15)	0.36	55.1	0.22–0.47	-
Griffon Bruxellois	2	50%	20	10%	0.13	22.6	0.15 (0.06)	24.2 (3.23)	-	0.11–0.64
Boxer	13	18%	4	50%	0.31 (0.06)	41.0 (5.85)	0.30 (0.07)	38.2 (8.42)	0.24–0.55	0.02–0.31
Shih Tzu	13	8%	7	43%	0.20 (0.07)	28.5 (3.25)	0.22 (0.05)	29.2 (5.00)	0.27–0.55	0.04–0.45
Chihuahua	5	40%	3	0%	0.34 (0.17)	20.0 (1.20)	0.41	19.2	0.01–0.05	0.02–0.26
CKCS	26	4%	11	18%	0.39 (0.07)	31.2 (4.85)	0.36 (0.05)	29.2 (4.10)	0.05–0.32	0.01–0.19
Affenpinscher	1	0%	31	10%	0.20	21.1	0.23 (0.08)	23.6 (4.30)	-	0.04–0.36
Staffordshire Bull Terrier	16	6%	2	0%	0.50 (0.07)	39.2 (4.92)	0.45 (0.02)	38.8 (6.72)	0.04–0.05	-

### Risk factors for BOAS

In study 1, as relative muzzle length increased, the proportion of dogs affected dropped steeply. Over 80% of dogs with CFR <0.1, i.e. the muzzle less than one tenth of the cranial length, were affected. In contrast, no dogs with CFRs of 0.5 or longer were affected; the affected dog with the longest muzzle was a Staffordshire Bull Terrier with a CFR of 0.49. There were 236 dogs with CFR values of 0.49 or less, falling within the conformationally ‘at-risk’ range: the relatively shortest to longest muzzle lengths affected by BOAS.

As well as shorter craniofacial ratios, increased absolute neck girths were significantly associated with greater BOAS risk. We determined statistical significance for both factors using generalised linear mixed models (GLMMs) for binary outcomes (including breed as a random effect) (Table 3). No other morphometric factors were significantly associated with BOAS risk in any models, including the presence of a nasal fold (P>0.05).

**Table 3:** Mixed model results for Studies 1 and 2 demonstrating risk factors for brachycephalic obstructive airway syndrome (BOAS). Body condition score and neuter status were non-significant in Study 1, and were excluded from the final model.

Variable	Study 1				Study 2			
	OR	SE	z	P	OR	SE	z	P
Intercept	4.36	0.89	1.65	0.09	0.0015	2.10	-3.09	0.002
Craniofacial ratio	0.000001	2.06	-7.85	<0.001	0.0000003	3.83	-3.95	<0.001
Neck girth	1.05	0.02	2.04	0.04	1.09	0.04	2.01	0.04
Body condition score	-	-	-	**-**	2.86	0.33	3.18	0.01
Neutered	-	-	-	-	5.66	0.70	2.48	0.01

**Fig 2:**
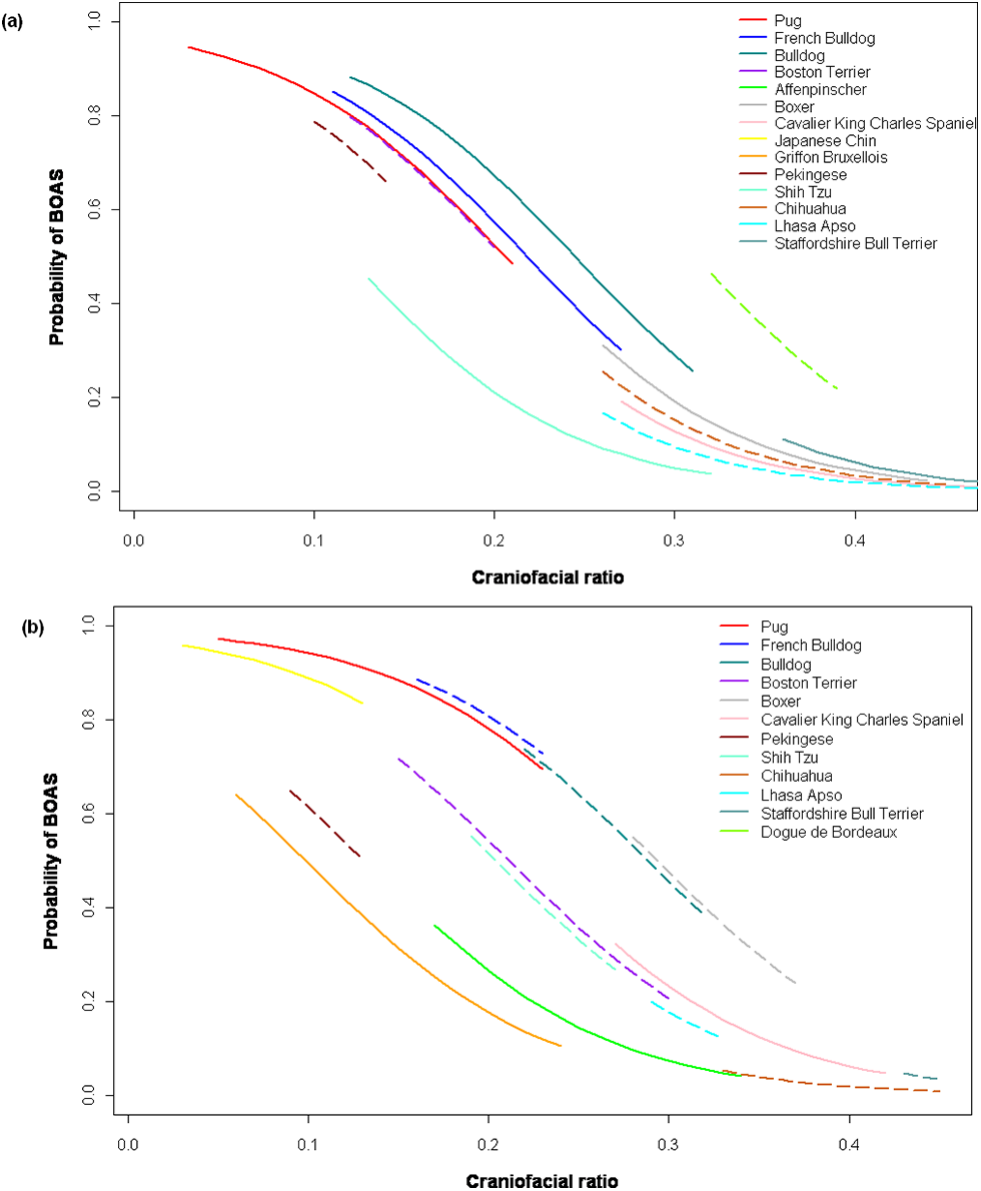
(a) (b) Predicted probability of brachycephalic dog breeds being affected by brachycephalic obstructive airway syndrome (BOAS) across relevant craniofacial ratio (CFR) and neck girth ranges. The risks across the CFR spectrum are calculated by breed using GLMM equations based on (a) Study 1 referral population data and (b) Study 2 non-referral population data. For each breed, the estimates are only plotted within the CFR ranges observed in the study populations. Dotted lines show breeds represented by <10 individuals. The breed mean neck girth is used for each breed (as stated in Table 2). In (b), the body condition score (BCS) = 5 (ideal bodyweight) and neuter status = neutered.

In line with policy requests for quantitative limits to conformational traits to be available for inclusion in Breed Standards [34,35], we used the GLMM model estimates to generate breed-specific predictions for BOAS risk across the CFR spectrum (Fig 2A; Table A in S1 File). For example, Pugs with the shortest observed CFR of 0.03 had a predicted BOAS risk of 0.95, compared with almost half that risk, 0.48, when the CFR was 0.21, the most moderate CFR for this breed (when neck girth was 32cm, the breed mean).

From the model predictions, the top three highest risk breeds were identified as the Pug, French Bulldog and Bulldog. When represented graphically, clear differences emerge between the different brachycephalic breeds with regard to BOAS risk (Fig 2A). Each curve incorporates information on each breed’s CFR range (length of line), random effect coefficient and mean neck girth.

## Materials and Methods–Study 2

### Morphometrics

Dogs were measured in an identical manner to Study 1.

### Recruitment of dogs

Between July 2012 and April 2013, brachycephalic dogs (n = 154) were recruited from non-referral populations for Study 2: breeders (79%), first opinion veterinary practice (14%), and rescue centres (7%). Inclusion criteria for this study were individual dogs:

1. With a craniofacial ratio <0.5 (as measured by RMAP). This value represents the longest relative muzzle length observed in an affected dog in Study 1.
2. That based on their temperament were suitable for handling for ~30 minutes and comfortable with a standard veterinary physical examination including rectal temperature measurement.

Both cross and pure breed brachycephalic dogs of all ages and gender were eligible for this study. To reflect the varied nature of the non-referral population, dogs were recruited from a variety of sources in the South East of England. These included:

1. Breeders. Dogs currently active in the breeding (and/or showing) population were considered of high priority for this study, as they have the most genetic influence on the next generation of their respective breed. Breed clubs of those breeds considered at high-risk due to their morphology (i.e. CFR<0.5), that were poorly represented or not included in the first study were targeted specifically. This included the Affenpinscher, Griffon Bruxellois, Pekingese, Japanese Chin and Tibetan Spaniel. Appropriate Breed clubs in the South East and London regions were contacted. In addition, to increase the number of all brachycephalic breeds that were currently used in breeding and showing, individual breeders in the South East and London were identified and contacted via the Kennel Club listings of members of the Assured Breeder Scheme.
2. Rehoming centres. This population was anticipated to incorporate several numerically large breeds in the overall UK canid population that had previously been underrepresented in the RVC SARH referral population. This includes the Staffordshire Bull Terrier (n = 16) and its crosses. This breed represented only 2.35% of the referral population sample, but represents the 2^nd^ most popular breed in the UK, based on microchip data, and 6^th^ based on Kennel Club rank in 2008 [52]. Two centres; Dogs Trust Salisbury and Kenilworth were visited to include dogs in the unowned brachycephalic population.
3. First opinion veterinary practice. This population was anticipated to incorporate brachycephalic dogs with non-referral level disorders, and healthy dogs, for example those presented for routine vaccinations. This population could potentially include dogs with mild BOAS that were not considered in need of further investigation or treatment. A large first opinion veterinary practice (Northolt, UK) volunteered as a study centre. Promotional material including posters to be displayed in the waiting room and consulting rooms were produced to advertise the study to relevant clients.

### Identification of BOAS cases

As diagnosis under general anaesthesia was not possible for these cases, close observation of clinical signs before and after a gentle exercise challenge was instead used to assess the presence and severity of BOAS. For example, the presence and intensity of ‘stertor’, a low-frequency snoring sound, can easily be recorded and, on fluoroscopy, the sound has previously been shown to correlate with transient inhalation of an elongated soft palate into the larynx [30].

To systematically assess BOAS clinical signs, a new standardised clinical examination protocol was therefore devised and carried out by the same assessor for all study dogs (MST):

1. The dog’s respiratory noise was noted at rest, and recorded for 30 seconds using an audio recording device (Sony ICD-UX200 Digital Voice Recorder and Sony ECM-CS10 Zoom Clip-Style Microphone), with the microphone attached to the dog’s collar, directly below the mouth.
2. Pre-exercise, the presence and frequency of the following clinical signs and behaviours were noted: open mouth breathing, dyspnoea, cyanotic mucous membranes, abnormal respiratory noise, choking and gagging episodes, yawning, postural adjustments e.g. stretched neck, nasal discharge and sneezing.
3. The presence of internal, referred respiratory noise was detected via auscultation using a recording stethoscope (Littmann 3200 Electronic 12 Track Stethoscope: 3200BU12) over the trachea (caudal to the larynx) and the thorax.
4. Temperature was measured rectally, pulse and respiratory rate were recorded and capillary refill time was assessed.
5. The dog was walked at a steady walking pace for 5 minutes. If at the RVC SARH this was over a set route around the hospital car park. If this was at a breeder’s residence, at a first opinion practice, or at a Dogs Trust centre, an appropriate 5 minute route was advised by the owner or dog’s carer. The walk was curtailed if the dog appearedto show excessive signs of respiratory distress.
6. On return from the walk, steps 1–4 were repeated in order to assess any change in respiration behaviour and physiology in comparison to pre-exercise (See Video A in S3 File)

Following this assessment, the veterinary assessor (MST) then answered the following question for each dog: In your professional opinion is this dog affected by BOAS? [YES]/[NO]

As syncope (collapse) was unlikely to be seen within this clinical examination, owners were questioned as to whether this had occurred in their dog’s history. All owners were questioned as to whether their dog had undergone any previous surgery to correct for BOAS, and to avoid incorrectly attributing clinical signs of other disorders to BOAS where possible, all owners were questioned regarding other known respiratory conditions in their dog.

As an ethical note, in dogs where a significant clinical problem was present, further investigation by a veterinarian was recommended to the owner, strongly so if the problems were severe (e.g. marked dyspnoea or history of syncope).

### Statistical analysis

Statistical analyses were performed in an identical manner to Study 1.

## Results–Study 2

### Population demographics and clinical status

In the brachycephalic non-referral population, 154 dogs represented 19 different breeds, with 94% pure bred. Of the 147 purebred dogs, 132 (90%) were Kennel Club registered. Female dogs were overrepresented (65%), with the majority of both sexes being entire (66%), reflecting the breeding dogs included in this population. The mean ± SE age was 4.12 ± 0.23 years, and mean ± SE weight (kg) was 9.44 ± 0.79, with 57% of dogs being overweight (BCS>5/9).

In total, 59 (38.3%) were categorised as affected by BOAS, representing 10 breeds (Table 2). Affected cross breeds included two Staffordshire Bull Terrier crosses (3%) and one Mastiff cross (2%). No owners reported that their dogs had undergone previous surgery to correct for BOAS.

### Risk factors for BOAS

In study 2, both shorter craniofacial ratios and thicker neck girths were confirmed to increase BOAS risk, as in study 1 (Table 3). In addition, a further two lifestyle factors were discovered that independently predicted an increased BOAS risk: being more overweight and being neutered. As before, we used GLMM model parameters to estimate breed-specific probabilities of BOAS across the CFR spectrum (Fig 2B and Table B in S1 File), revealing marked similarities between model estimates from Studies 1 and 2. From the model predictions, the highest risk breeds were again identified as the Pug, French Bulldog and Bulldog.

The predicted effects of neck girth and body condition were relatively subtle compared with CFR (Figure A in S2 File). To demonstrate the effect of neck girth, a neutered Pug with the breed average CFR (0.11) and neck girth (32 cm) had a predicted BOAS risk of 0.93; however, if neck girth was reduced by 5cm the predicted risk decreased slightly to 0.89. Considering body condition in the same ‘average’ Pug, if BCS increased by just 1 point to 6 (slightly overweight), the predicted risk rose from 0.93 to 0.98.

### Study comparison

Model predictions were markedly similar between the two populations. To demonstrate this similarity, for a dog of non-specified breed with a neck girth of 20cm, at CFR 0.1, the predicted BOAS probability was 0.69 for Study 1, compared with 0.68 for Study 2. Likewise, for CFR 0.2, the predicted BOAS probability for Study 1 was 0.31, and for Study 2 it was 0.32.

### Conformation vs breed affiliation

**Fig 3:**
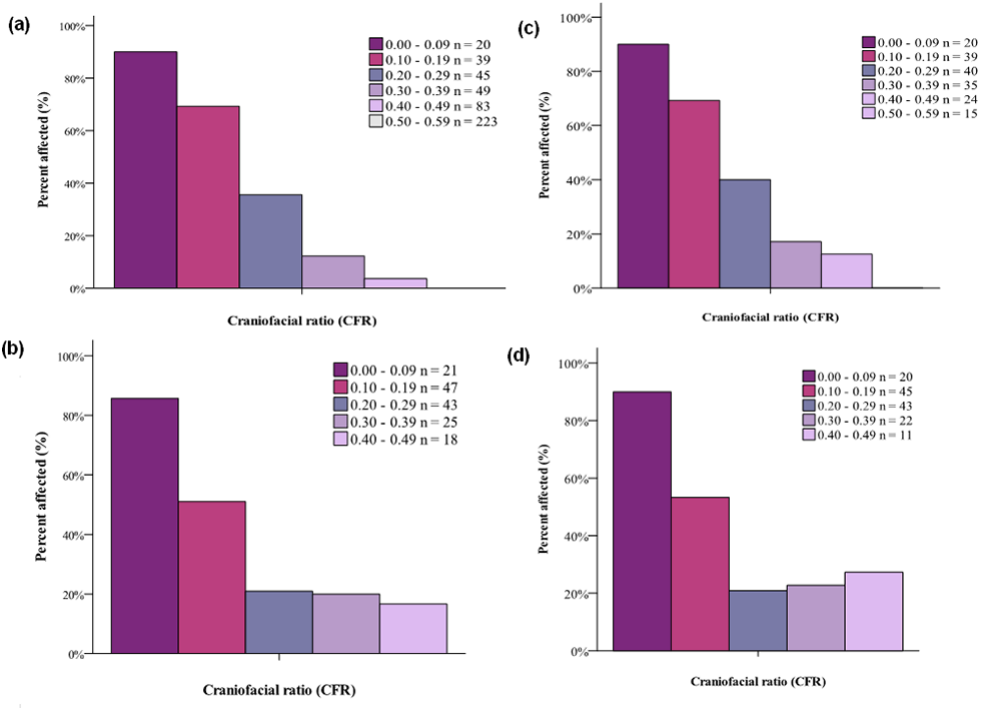
Percentage of dogs affected by BOAS by craniofacial ratio (CFR) category (a-d). Graphs (a) and (c) represent all dogs in populations 1 (n = 700) and 2 (n = 154), respectively. Graphs (b) and (d) only represent dogs of breeds and their crosses that were affected by BOAS in population 1 (n = 174) and 2 (n = 141), respectively. The breed-restricted population demonstrates the effects of conformation whilst keeping the genetic and environmental background as uniform as possible. The marked risk of CFRs <0.20 is clearly demonstrated in both studies, with >50% of dogs affected.

Fig 3 shows that flat facial conformation, aside from other aspects of breed affiliation, plays a major role in determining whether or not a dog has BOAS. The drop in the proportion of affected dogs with higher CFRs was steep regardless of whether all breeds were considered (Fig 3A and 3C), or just those breeds and crossbreeds comprising affected individuals, i.e. when we limited the genetic background solely to affected breeds (Fig 3B and 3D). The direct effect of facial conformation is all the more evident in Fig 3, because the relationship persists despite the fact that not all brachycephalic breeds are closely related to each other [53]. This gives little reason to suppose they would share a genetic trait predisposing them to BOAS by common descent, other than brachycephaly itself.

## Discussion

Our results confirm that brachycephaly is a risk factor for BOAS and for the first time quantitatively demonstrate that more extreme brachycephalic conformations are at higher risk of BOAS than more moderate morphologies; BOAS risk increases sharply in a non-linear manner as relative muzzle length shortens. Although susceptibility varies greatly between breeds (Fig 2A and 2B), these results suggest that breeding towards relatively longer CFRs may indeed reduce BOAS risk within affected breeds. In addition to this finding, increased neck girth and obesity were found to be further exacerbating factors for BOAS. Despite being studied in two different populations, the results showed marked similarities, indicating the strong biological nature of the relationship between morphology and disease.

This study has enabled the first quantitative estimates associating conformation with the risk of BOAS, as requested by the Council of Europe (1995). We have demonstrated the association across a variety of breeds, independent of breed-genetic factors, which were accounted for through the random effect of breed in the mixed model analysis. As such, general risk factor estimates have been modelled, that can be used to guide breeding decisions. Specific estimates have also been modelled for a variety of high-risk brachycephalic breeds. These results can be used to introduce quantitative limits to the degree of brachycephaly prescribed by breed standards, to encourage breeding for more moderate craniofacial morphology, in order to reduce the prevalence and severity of BOAS. Interestingly, the three most affected breeds in both of our studies are the same as the three most affected in a previous study on a Belgian referral population [11]. Breeders of these breeds and others can use these estimates to see how high-risk their current breeding stock are, and plan matings utilising this information to reduce BOAS risk of in future offspring by aiming for safer conformations. Stakeholders can now consider these values and decide what they believe is an ‘acceptable’ disease-risk.

### Brachycephaly as a risk factor for BOAS

As asserted by Oechtering (2010), there are ‘inevitable’ major consequences of markedly reducing the bony framework of an organ [54]. Our results show that breeding for extreme craniofacial morphology results has a profound effect upon the risk of obstructive upper airway disease in domestic dogs. This scaling effect had not previously been demonstrated, with little objective evidence that more extreme brachycephalic dogs were at increased risk [11]. The particularly strong relationship between BOAS and brachycephalic phenotypes suggests that, although BOAS susceptibility varies between breeds (Fig 2), breeding away from extreme brachycephaly would reduce BOAS risk generally. That said, whether breeds can feasibly be selectively bred for lower risk morphologies relies on there being sufficient existing phenotypic variability in the breed. That is not always the case, e.g. the highest CFR in the Japanese Chin in our studies was just 0.13, with an associated predicted BOAS probability of 0.83. Judicious out-crossing to introduce ‘safe’ conformations into a breed is controversial, but might be required on the basis of animal welfare in populations with extremely high prevalences of BOAS and limited morphological variation.

Foreshortened CFR is the key observable trait that has been selected for in the genetically complex brachycephalic conformation [55–57], and it is likely that this external measure is a proxy for aspects of internal morphology that skeletal foreshortening has had a pathological impact upon. Reduced facial bone morphology without concurrent and proportional reduction of the structures held within it is likely to have led to relative elongation and thickening of the soft palate, and relative oversize of the turbinates. Additional internal factors may predispose individuals or specific breeds to increased airway resistance e.g. a low glottic index [58] and angles of the internal facial bones e.g. the hard palate. It is possible that each breed has different internal risk factors that are incorporated within their random effect statistic in the models presented here, causing them to have differing risks of BOAS. Further study of the internal morphology of the airways could investigate the relationship between these internal structures and external muzzle foreshortening. Nevertheless, from an applied point of view, the CFR is an important variable because dog breeders and buyers will more easily be able to select healthy dogs on the basis of their externally visible conformation.

Despite the strong relationship between CFR and BOAS risk, it is likely that other genetic and environmental factors will also contribute to the likelihood and severity of BOAS in individual dogs. We found some exceptional individuals that were unaffected by BOAS despite extreme brachycephaly (e.g. 18 individuals in Study 1, and 29 in Study 2, appeared unaffected despite having CFRs <0.2), that may have anatomical and/or physiological adaptations. Brachycephaly has concurrently led to a ‘space problem’ for the canine brain [59], with anatomical changes observed in brachycephalic dogs such as ventral pitching of the primary longitudinal brain axis, a ventral shift in the position of the olfactory lobe [59], and more perpendicular development of the cranium relative to the facial axis [60] hypothesised to be adaptations representing a biological solution to this problem. It is possible that such anatomical adaptations to solve the airway ‘space problem’ associated with brachycephaly exist, and may be selectable via respiratory assessment, but this requires further investigation of exceptional individuals.

Due to ethical and financial limitations not allowing for internal airway examination of all study dogs, BOAS was diagnosed in study 1 based on the nares ratio and ORB score. It is possible that some dogs with BOAS may have lacked stenotic nares but had other airway abnormalities (e.g. an elongated soft palate) and a high ORB score, but were classified as unaffected. Further study of BOAS populations could identify whether certain conformations are associated with specific features of BOAS or not, to ascertain the extent to which this may have affected the results presented here. Regarding diagnosis in Study 2, it is possible that owners may have been dishonest with their answers regarding previous surgeries to correct for BOAS, which may have led to some dogs being erroneously classed as unaffected by BOAS. In addition, as some dogs were recruited from rescue centres, full veterinary histories may not be available. Although clinical signs can improve post-surgically, many ‘corrected’ dogs are not considered ‘normal’ [45], and may still be restricted in their activities compared with unaffected animals, thus may still be assessed as affected. As dogs were diagnosed without direct visualisation of the pharynx, it is possible that stertor was caused by other pathologies; however, as these can be assumed to be unrelated to cranial conformation, this would have reduced our ability to detect a relationship between conformation and classification as affected by BOAS.

### Neck girth as a risk factor for BOAS

Thick neck girth and obesity have not previously been implicated in BOAS; however, both are risk factors for OSAS in humans. Thick neck circumference predicts OSAS more effectively than measures of obesity (e.g. BMI, waist: hip ratio) [46–50]. It is unclear to what degree neck girth is independent of obesity in dogs, or to what extent environmental and genetic factors contribute. If there is a strong genetic component to neck girth, selective breeding away from thicker necks would help combat BOAS. In several high-risk Kennel Club breeds a ‘thick’ neck is explicitly encouraged [61–63]. This is additionally encouraged by the American Kennel Club in the Pug and French Bulldog [64,65], with more extreme references to a ‘very thick’ neck in the Bulldog [66]. This may have led to artificial selection for disproportionate fat deposition in this region. As such, breed-specific quantitative data outlining maximum neck girth values may be a further strategy to reduce BOAS risk.

Neck girth was only a significant predictor of BOAS when included as an absolute measure, and not when normalised against other measurements (including width of the head and chest, chest girth and PC1). It is important to remember that the models included multiple predictors, so neck girth was significant in the context of all the other predictors in the model, i.e. once CFR, Breed and other factors were also taken into account. Thus, the models suggest that absolute neck girth is important *for a given breed with a given CFR*. It is possible that the absolute weight of the tissue in the neck, rather than the relative proportions of it to the airway, has an effect on its capacity to compromise the airway. That is, the same relative neck girth might differ in its capacity to impinge on relevant airway structures. This is also seen in human OSAS, with an absolute neck circumference greater than 16 inches in a woman or greater than 17 inches in a man correlating with an increased risk for the disorder [46], with increasing neck girth correlating with the severity of apnoea [48]. It should be noted that neck girth was a relatively small effect when compared to CFR, and thus a high neck girth alone may not be sufficient to cause BOAS. Further study of the necks of brachycephalic dogs, including dynamic CT, may elucidate the role of neck girth in airway compromise [67].

### Obesity as a risk factor for BOAS

Regarding obesity, generalised adipose tissue deposition could well include the palate, tongue and tissues surrounding the airways. Indeed, obesity narrows the pharyngeal airway in Zucker rats, and increases upper airway collapsibility [68]. Furthermore, in humans, weight loss can alleviate OSAS [69], so maintaining a lean body condition in dogs with BOAS is potentially important. Obesity may have further general effects on the respiratory system of the dog, with barometric whole body plethysmography demonstrating that obese dogs had a significantly decreased tidal volume (per kg) and significantly increased respiratory rate [70] compared with non-obese dogs. Whether obesity is a causal factor in the development of BOAS, or an exacerbating factor for an existing case cannot be elucidated from our data. Indeed, it is possible that high body condition scores in BOAS affected dogs are a consequence of BOAS limiting their abilities to exercise and thus increasing their likelihood of obesity. A longitudinal study is required to determine whether dogs became obese before or after exhibiting BOAS clinical signs [71]. Obesity is prevalent in the general canine population, with an estimated 20–40% of dogs affected [72]. In the non-referral population of brachycephalic dogs, 56.5% of dogs were overweight with a BCS of over 5, and 10% of dogs even had a BCS of over 7. Due to the potential exacerbating effects upon BOAS, keeping dogs at a healthy body weight is advocated.

### Neutering

Neutering as a risk factor for BOAS is also a novel finding in Study 2. It is possible that this is not a causal biological effect; rather, it could be a circular association, with BOAS causing withdrawal from breeding and showing (thus leading to neutering) in show-dogs due to rules in place to discourage unhealthy dogs in the showing and breeding population (e.g. the Kennel Club initiative ‘Breed Watch’ warns against breathing difficulties in several brachycephalic breeds). As such, neutering becomes associated with BOAS in the show and breeding-dog heavy population of Study 2. This would then lead to a disproportionate number of BOAS affected dogs being neutered, and would be an encouraging sign of effort already being made in the UK breeding community to avoid breeding from affected dogs. This finding may also be an artefact of the unusual population studied here; outside of the veterinary environment, and with a high percentage of show dogs included. If this were to be a biological effect, explanations for this effect include neutering being a risk factor for obesity [73], which was also found to be a risk factor for BOAS. Effects of sex hormones on the respiratory system appear an unlikely cause. As such, from these data, keeping dogs entire is not advocated as part of the prevention strategy for BOAS in individual dogs; however, neutering dogs affected by BOAS may be helpful to avoid its perpetuation in future generations. Although unlikely to be widely adopted without policy level intervention, veterinarian Harvey [12] insisted that if dogs are to undergo BOAS surgery, then they must also be neutered at the same time.

### Reducing BOAS risk

If society wanted to eliminate BOAS from the domestic dog population entirely then based on these data a quantitative limit of CFR no less than 0.5 (approximately describing the CFR of an average Staffordshire Bull Terrier) would need to be imposed. However, with the current popularity of brachycephalic dogs both in the UK and internationally [74], this is unlikely to be implemented as it would require the cessation of breeding of many popular breeds. Also it would unnecessarily exclude breeding from many moderately brachycephalic dogs that were actually free of BOAS. Even if society were to decide that, say, a 50% risk of BOAS were acceptable, this would mean ceasing to breed from dogs with a CFR of around 0.2 or less, approximately describing an average French Bulldog. Many breeds include few if any individuals with CFRs above this threshold, so they would struggle to survive such a policy and the breeds might effectively be ‘banned’. Banning is controversial, with a recent survey finding approximately as many UK stakeholders in favour of banning affected breeds immediately as those entirely against banning [37].

If society wanted to reduce BOAS risk, but not ban any existing breeds, then an even more moderate strategy could be adopted. Several approaches could be used towards breeding towards more moderate, lower-risk morphologies, each of which may have strengths and weaknesses and may be differentially supported by stakeholders involved in this issue [37]:

1. Selecting only those dogs with more moderate, lower-risk morphologies for breeding. The further amendment of breed standards to promote lower-risk morphologies and penalise high-risk, extreme morphologies (potentially including quantitative limits) may aid this approach,
2. Health screening of morphologically extreme dogs to help select only those that are free of BOAS for breeding,
3. Developing genetic tests to highlight high and low risk animals e.g. individuals within high risk breeds with or without elongated soft palates. Genetically testing for the anatomical abnormalities of BOAS may not be the optimal solution as these features may be strongly linked with skull morphology, and thus this may not be feasible strategy
  and/or
4. For breeds lacking sufficient individuals with moderate morphologies, judicious out-crossing to increase health and phenotypic diversity. This approach would require the necessary cooperation from kennel clubs.

## Conclusions

We present an example of how focussed selective breeding for one desired extreme morphology can result in an unintentional pathology detrimental to animal welfare. Our results quantitatively demonstrate for the first time how breeding for flatter faces dramatically increases the risk of chronic airway obstruction in domestic dogs. The phenomenon of a desired conformation directly impacting on normal function is likely to extend to all domesticated species with malleable phenotypes. As such, this study not only has implications for dog breeding in the UK, but domestic animal breeding programmes internationally. Although relaxed functional demands may have facilitated diversification of companion dogs’ skull morphology [33], health should be one of the primary considerations when breeding companion animals.

## Supporting Information

S1 File Predicted probabilities of being affected by brachycephalic obstructive airway syndrome (BOAS) by breed across the brachycephalic craniofacial ratio (CFR) spectrum. Data is presented from Study 1 (referral population) data (**Table A in S1 File**) and Study 2 (non-referral population) data (**Table B in S1 File**).
(DOCX)

S2 File Predicted probability of a dog being affected by brachycephalic obstructive airway syndrome (BOAS) across the brachycephalic craniofacial ratio (CFR) for three neck girths. Data presented from Study 1 (**Figure Aa in S2 File**) and Study 2 (**Figure Ab in S2 File**).
(DOCX)

S3 File Video of signs of brachycephalic obstructive airway syndrome (BOAS). (DOCX)

S1 Dataset Raw data from study 1. (XLSX)

S2 Dataset Raw data from study 2. (XLSX)
