# Supplementary material for: Impact of Facial Conformation on Canine Health: Brachycephalic Obstructive Airway Syndrome
Source: PLoS One. 2015 Oct 28;10(10):e0137496. doi: 10.1371/journal.pone.0137496 (PMC4624979; doi:10.1371/journal.pone.0137496)
Supplement: S1 File — Data is presented from Study 1 (referral population) data (Table A in S1 File) and Study 2 (non-referral population) data (Table B in S1 File). (DOCX) [file pone.0137496.s001.docx]

**S1 File. Predicted probabilities of being affected by brachycephalic obstructive airway syndrome (BOAS) by breed across the brachycephalic craniofacial ratio (CFR) spectrum.** Data is presented from Study 1 (referral population) data (**Table A**) and Study 2 (non-referral population) data (**Table B**).

**S1 Table A. Predicted probability of being affected by brachycephalic obstructive airway syndrome (BOAS) by breed across the brachycephalic craniofacial ratio (CFR) spectrum, from Study 1 (referral population) data.** The probabilities are calculated using GLMM parameters outlined in Table 2 combined with the random effect coefficient for each breed. Only the probabilities associated with CFR values observed in this population for each breed are stated, with the breed mean CFR indicated by an emboldened value in a highlighted box. Red indicates P= 0.8-1.0, Orange=0.6-0.79, Yellow=0.4-0.59, Light Yellow=0.2-0.39 and Light Green=0.0-0.19. The breed mean neck girth is used for each breed (as stated in Table 2), BCS=5 (ideal bodyweight) and Neuter status=Neutered.

| **Craniofacial Ratio** | **Probability (P) of BOAS** | | | | | | | | | | |
| --- | --- | --- | --- | --- | --- | --- | --- | --- | --- | --- | --- |
|  | **Pug** | **Pekingese** | **French Bulldog** | **Boston Terrier** | **Bulldog** | **Shih Tzu** | **Boxer** | **Chihuahua** | **Cavalier King Charles Spaniel** | **Dogue de Bordeaux** | **Staffordshire Bull Terrier** |
| 0.03 | 0.95 |  |  |  |  |  |  |  |  |  |  |
| 0.04 | 0.94 |  |  |  |  |  |  |  |  |  |  |
| 0.05 | 0.93 |  |  |  |  |  |  |  |  |  |  |
| 0.06 | 0.91 |  |  |  |  |  |  |  |  |  |  |
| 0.07 | 0.90 |  |  |  |  |  |  |  |  |  |  |
| 0.08 | **0.88** |  |  |  |  |  |  |  |  |  |  |
| 0.09 | 0.87 |  |  |  |  |  |  |  |  |  |  |
| 0.1 | 0.85 | 0.79 |  |  |  |  |  |  |  |  |  |
| 0.11 | 0.83 | 0.76 | 0.85 |  |  |  |  |  |  |  |  |
| 0.12 | 0.80 | **0.73** | 0.83 | 0.80 | 0.88 |  |  |  |  |  |  |
| 0.13 | 0.77 | 0.70 | 0.81 | 0.77 | 0.86 | 0.45 |  |  |  |  |  |
| 0.14 | 0.74 | 0.66 | 0.78 | 0.74 | 0.84 | 0.41 |  |  |  |  |  |
| 0.15 | 0.71 |  | 0.75 | **0.71** | 0.82 | 0.38 |  |  |  |  |  |
| 0.16 | 0.68 |  | 0.72 | 0.67 | 0.80 | 0.34 |  |  |  |  |  |
| 0.17 | 0.64 |  | 0.69 | 0.64 | 0.77 | 0.30 |  |  |  |  |  |
| 0.18 | 0.60 |  | 0.65 | 0.60 | 0.74 | 0.27 |  |  |  |  |  |
| 0.19 | 0.56 |  | **0.61** | 0.56 | 0.71 | 0.24 |  |  |  |  |  |
| 0.2 | 0.52 |  | 0.57 | 0.52 | 0.67 | **0.21** |  |  |  |  |  |
| 0.21 | 0.48 |  | 0.53 |  | 0.64 | 0.19 |  |  |  |  |  |
| 0.22 |  |  | 0.49 |  | **0.60** | 0.16 |  |  |  |  |  |
| 0.23 |  |  | 0.45 |  | 0.56 | 0.14 |  |  |  |  |  |
| 0.24 |  |  | 0.41 |  | 0.52 | 0.12 |  |  |  |  |  |
| 0.25 |  |  | 0.37 |  | 0.48 | 0.11 |  |  |  |  |  |
| 0.26 |  |  | 0.34 |  | 0.44 | 0.09 | 0.31 | 0.26 |  |  |  |
| 0.27 |  |  | 0.30 |  | 0.40 | 0.08 | 0.28 | 0.23 | 0.19 |  |  |
| 0.28 |  |  |  |  | 0.36 | 0.07 | 0.25 | 0.20 | 0.17 |  |  |
| 0.29 |  |  |  |  | 0.32 | 0.06 | 0.22 | 0.17 | 0.15 |  |  |
| 0.3 |  |  |  |  | 0.29 | 0.05 | 0.19 | 0.15 | 0.13 |  |  |
| 0.31 |  |  |  |  | 0.26 | 0.04 | 0.17 | 0.13 | 0.11 |  |  |
| 0.32 |  |  |  |  |  | 0.04 | **0.15** | 0.12 | 0.10 | 0.47 |  |
| 0.33 |  |  |  |  |  |  | 0.13 | 0.10 | 0.08 | 0.43 |  |
| 0.34 |  |  |  |  |  |  | 0.11 | 0.09 | 0.07 | 0.39 |  |
| 0.35 |  |  |  |  |  |  | 0.10 | **0.07** | 0.06 | 0.35 |  |
| 0.36 |  |  |  |  |  |  | 0.08 | 0.06 | 0.05 | **0.31** | 0.11 |
| 0.37 |  |  |  |  |  |  | 0.07 | 0.05 | 0.05 | 0.28 | 0.10 |
| 0.38 |  |  |  |  |  |  | 0.06 | 0.05 | 0.04 | 0.25 | 0.08 |
| 0.39 |  |  |  |  |  |  | 0.05 | 0.04 | 0.03 | 0.22 | 0.07 |
| 0.4 |  |  |  |  |  |  | 0.04 | 0.03 | **0.03** |  | 0.06 |
| 0.41 |  |  |  |  |  |  | 0.04 | 0.03 | 0.02 |  | 0.05 |
| 0.42 |  |  |  |  |  |  | 0.03 | 0.03 | 0.02 |  | 0.05 |
| 0.43 |  |  |  |  |  |  | 0.03 | 0.02 | 0.02 |  | 0.04 |
| 0.44 |  |  |  |  |  |  | 0.02 | 0.02 | 0.01 |  | 0.03 |
| 0.45 |  |  |  |  |  |  |  | 0.02 | 0.01 |  | 0.03 |

| **Colour** | **CFR** | **Risk** |
| --- | --- | --- |
|  | 0.8-1.0 | Extremely high risk |
|  | 0.6-0.79 | High risk |
|  | 0.4-0.59 | Medium risk |
|  | 0.2-0.39 | Increased risk |
|  | 0.0-0.19 | Low risk |

**S1 Table B. Predicted probability of being affected by brachycephalic obstructive airway syndrome (BOAS) by breed across the brachycephalic craniofacial ratio (CFR) spectrum, from Study 2 (non-referral population) data.** The probabilities are calculated using GLMM parameters outlined in Table 2 combined with the random effect coefficient for each breed. Only the probabilities associated with CFR values observed in this population for each breed are stated, with the breed mean CFR indicated by an emboldened value in a highlighted box. Red indicates P= 0.8-1.0, Orange=0.6-0.79, Yellow=0.4-0.59, Light Yellow=0.2-0.39 and Light Green=0.0-0.19. The breed mean neck girth is used for each breed (as stated in Table 2), BCS=5 (ideal bodyweight) and Neuter status=Neutered.

| **Craniofacial Ratio**  **(CFR)** | **Probability (P) of BOAS** | | | | | | | | | | | | | |
| --- | --- | --- | --- | --- | --- | --- | --- | --- | --- | --- | --- | --- | --- | --- |
|  | **Japanese Chin** | **Pug** | **Griffon Bruxellois** | **Pekingese** | **Boston Terrier** | **French Bulldog** | **Affenpinscher** | **Shih Tzu** | **Bulldog** | **Cavalier King Charles Spaniel** | **Boxer** | **Dogue de Bordeaux** | **Chihuahua** | **Staffordshire Bull Terrier** |
| 0.03 | 0.96 |  |  |  |  |  |  |  |  |  |  |  |  |  |
| 0.04 | 0.95 |  |  |  |  |  |  |  |  |  |  |  |  |  |
| 0.05 | 0.94 | 0.97 |  |  |  |  |  |  |  |  |  |  |  |  |
| 0.06 | **0.94** | 0.97 | 0.64 |  |  |  |  |  |  |  |  |  |  |  |
| 0.07 | 0.93 | 0.96 | 0.61 |  |  |  |  |  |  |  |  |  |  |  |
| 0.08 | 0.92 | 0.96 | 0.57 |  |  |  |  |  |  |  |  |  |  |  |
| 0.09 | 0.9 | 0.95 | 0.53 | 0.65 |  |  |  |  |  |  |  |  |  |  |
| 0.1 | 0.89 | 0.94 | 0.49 | 0.61 |  |  |  |  |  |  |  |  |  |  |
| 0.11 | 0.87 | **0.93** | 0.46 | **0.58** |  |  |  |  |  |  |  |  |  |  |
| 0.12 | 0.86 | 0.92 | 0.42 | 0.54 |  |  |  |  |  |  |  |  |  |  |
| 0.13 | 0.84 | 0.91 | 0.38 | 0.5 |  |  |  |  |  |  |  |  |  |  |
| 0.14 |  | 0.9 | 0.35 |  |  |  |  |  |  |  |  |  |  |  |
| 0.15 |  | 0.88 | **0.31** |  | 0.72 |  |  |  |  |  |  |  |  |  |
| 0.16 |  | 0.87 | 0.28 |  | 0.69 | 0.89 |  |  |  |  |  |  |  |  |
| 0.17 |  | 0.85 | 0.25 |  | 0.65 | 0.87 | 0.36 |  |  |  |  |  |  |  |
| 0.18 |  | 0.83 | 0.23 |  | 0.62 | 0.85 | 0.33 |  |  |  |  |  |  |  |
| 0.19 |  | 0.81 | 0.2 |  | 0.58 | **0.83** | 0.3 | 0.55 |  |  |  |  |  |  |
| 0.2 |  | 0.78 | 0.18 |  | 0.54 | 0.81 | 0.27 | 0.51 |  |  |  |  |  |  |
| 0.21 |  | 0.75 | 0.16 |  | 0.51 | 0.78 | 0.24 | 0.48 |  |  |  |  |  |  |
| 0.22 |  | 0.73 | 0.14 |  | 0.47 | 0.76 | 0.21 | **0.44** | 0.74 |  |  |  |  |  |
| 0.23 |  | 0.69 | 0.12 |  | **0.43** | 0.73 | 0.19 | 0.4 | 0.71 |  |  |  |  |  |
| 0.24 |  |  | 0.11 |  | 0.39 |  | 0.17 | 0.37 | 0.68 |  |  |  |  |  |
| 0.25 |  |  |  |  | 0.36 |  | **0.15** | 0.33 | 0.64 |  |  |  |  |  |
| 0.26 |  |  |  |  | 0.32 |  | 0.13 | 0.3 | **0.61** |  |  |  |  |  |
| 0.27 |  |  |  |  | 0.29 |  | 0.11 | 0.27 | 0.57 | 0.32 |  |  |  |  |
| 0.28 |  |  |  |  | 0.26 |  | 0.1 |  | 0.53 | 0.29 | 0.55 |  |  |  |
| 0.29 |  |  |  |  | 0.23 |  | 0.08 |  | 0.49 | 0.26 | 0.51 |  |  |  |
| 0.3 |  |  |  |  | 0.21 |  | 0.07 |  | 0.46 | 0.23 | 0.48 |  |  |  |
| 0.31 |  |  |  |  |  |  | 0.06 |  | 0.42 | 0.21 | **0.44** |  |  |  |
| 0.32 |  |  |  |  |  |  | 0.06 |  | 0.38 | 0.18 | 0.4 | 0.47 |  |  |
| 0.33 |  |  |  |  |  |  | 0.05 |  |  | 0.16 | 0.37 | 0.43 | 0.05 |  |
| 0.34 |  |  |  |  |  |  | 0.04 |  |  | 0.14 | 0.33 | 0.39 | 0.05 |  |
| 0.35 |  |  |  |  |  |  |  |  |  | **0.12** | 0.3 | 0.35 | 0.04 |  |
| 0.36 |  |  |  |  |  |  |  |  |  | 0.11 | 0.27 | **0.31** | 0.03 |  |
| 0.37 |  |  |  |  |  |  |  |  |  | 0.1 | 0.24 | 0.28 | 0.03 |  |
| 0.38 |  |  |  |  |  |  |  |  |  | 0.08 |  | 0.25 | 0.03 |  |
| 0.39 |  |  |  |  |  |  |  |  |  | 0.07 |  | 0.22 | 0.02 |  |
| 0.4 |  |  |  |  |  |  |  |  |  | 0.06 |  |  | **0.02** |  |
| 0.41 |  |  |  |  |  |  |  |  |  | 0.05 |  |  | 0.02 |  |
| 0.42 |  |  |  |  |  |  |  |  |  | 0.05 |  |  | 0.01 |  |
| 0.43 |  |  |  |  |  |  |  |  |  |  |  |  | 0.01 | 0.05 |
| 0.44 |  |  |  |  |  |  |  |  |  |  |  |  | 0.01 | 0.04 |
| 0.45 |  |  |  |  |  |  |  |  |  |  |  |  | 0.01 | 0.04 |

| **Colour** | **CFR** | **Risk** |
| --- | --- | --- |
|  | 0.8-1.0 | Extremely high risk |
|  | 0.6-0.79 | High risk |
|  | 0.4-0.59 | Medium risk |
|  | 0.2-0.39 | Increased risk |
|  | 0.0-0.19 | Low risk |
