# Supplementary material for: Impact of Facial Conformation on Canine Health: Brachycephalic Obstructive Airway Syndrome
Source: PLoS One. 2015 Oct 28;10(10):e0137496. doi: 10.1371/journal.pone.0137496 (PMC4624979; doi:10.1371/journal.pone.0137496)
Supplement: S2 File — Data presented from Study 1 (Figure Aa in S2 File) and Study 2 (Figure Ab in S2 File). (DOCX) [file pone.0137496.s002.docx]

**S2 File. Predicted probability of a dog being affected by brachycephalic obstructive airway syndrome (BOAS) across the brachycephalic craniofacial ratio (CFR) for three neck girths.** Data presented from Study 1 (**Figure Aa**) and Study 2 (**Figure Ab**).

**S2 Figure A (a) (b) Predicted probability of a dog being affected by brachycephalic obstructive airway syndrome (BOAS) across the brachycephalic craniofacial ratio (CFR) for three neck girths.** To demonstrate the effect of varying neck girths (20, 30 and 40cm) on BOAS risk across the CFR spectrum, generalised (i.e. breed independent) estimates are calculated using GLMM equations based on (a) Study 1 referral population data and (b) Study 2 non-referral population data.


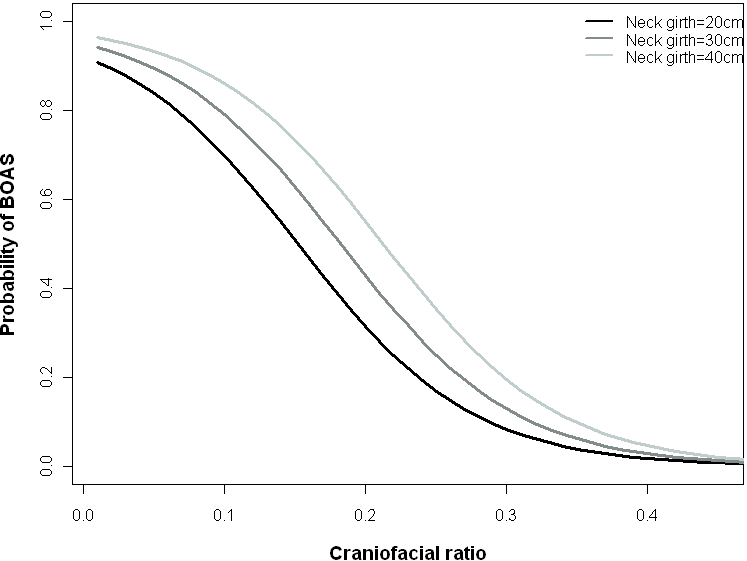


(a)


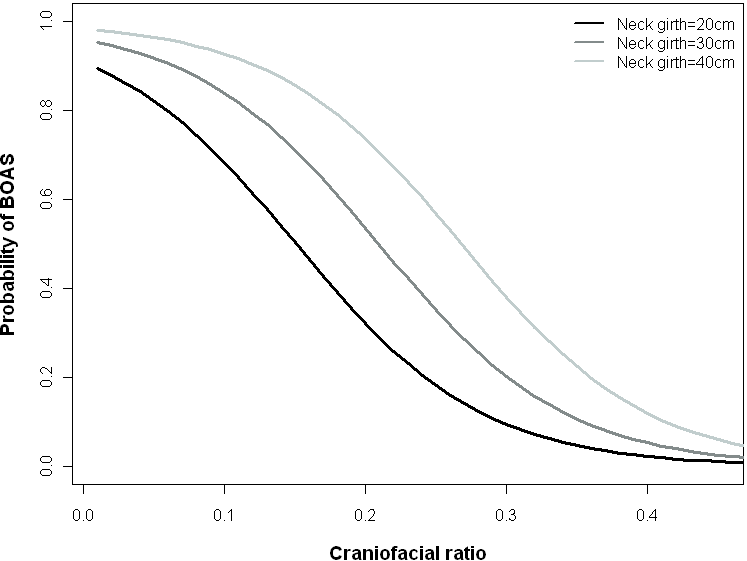
(b)
