# Supplementary material for: Impact of Facial Conformation on Canine Health: Brachycephalic Obstructive Airway Syndrome
Source: PLoS One. 2015 Oct 28;10(10):e0137496. doi: 10.1371/journal.pone.0137496 (PMC4624979; doi:10.1371/journal.pone.0137496)
Supplement: S3 File — (DOCX) [file pone.0137496.s003.docx]

**S3 File. Video of signs of brachycephalic obstructive airway syndrome (BOAS).**

**S3 Video A.** **Video of signs of brachycephalic obstructive airway syndrome (BOAS) in a two year old entire female Pug at home after 5 minutes of gentle walking.** The opportunity to walk did not include any forced locomotion. This dog shows nearly continuous stertor, open mouth breathing and increased respiratory effort. This individual had not been formally diagnosed with BOAS, but was classed as affected following the examination protocol used in Study 2. For this dog, CFR=0.12, neck girth=34cm and nares ratio=0.12.
